# Acute vestibular syndrome: is skew deviation a central sign?

**DOI:** 10.1007/s00415-021-10692-6

**Published:** 2021-07-09

**Authors:** Athanasia Korda, Ewa Zamaro, Franca Wagner, Miranda Morrison, Marco Domenico Caversaccio, Thomas C Sauter, Erich Schneider, Georgios Mantokoudis

**Affiliations:** 1 Department of Otorhinolaryngology, Head and Neck Surgery, Inselspital, University Hospital Bern and University of Bern, Bern, Switzerland; 2 University Institute of Diagnostic and Interventional Neuroradiology, Inselspital, University Hospital Bern and University of Bern, Bern, Switzerland; 3 Department of Emergency Medicine, Inselspital, University Hospital Bern and University of Bern, Bern, Switzerland; 4 Institute of Medical Technology, Brandenburg University of Technology Cottbus - Senftenberg, Senftenberg, Germany

**Keywords:** Test of skew, Acute unilateral vestibulopathy, Acute stroke, Vertigo, Video-oculography, VOG

## Abstract

**Objective:**

Skew deviation results from a dysfunction of the graviceptive pathways in patients with an acute vestibular syndrome (AVS) leading to vertical diplopia due to vertical ocular misalignment. It is considered as a central sign, however, the prevalence of skew and the accuracy of its test is not well known .

**Methods:**

We performed a prospective study from February 2015 until September 2020 of all patients presenting at our emergency department (ED) with signs of AVS. All patients underwent clinical HINTS and video test of skew (vTS) followed by a delayed MRI, which served as a gold standard for vestibular stroke confirmation.

**Results:**

We assessed 58 healthy subjects, 53 acute unilateral vestibulopathy patients (AUVP) and 24 stroke patients. Skew deviation prevalence was 24% in AUVP and 29% in strokes. For a positive clinical test of skew, the cut-off of vertical misalignment was 3 deg with a very low sensitivity of 15% and specificity of 98.2%. The sensitivity of vTS was 29.2% with a specificity of 75.5%.

**Conclusions:**

Contrary to prior knowledge, skew deviation proved to be more prevalent in patients with AVS and occurred in every forth patient with AUVP. Large skew deviations (> 3.3 deg), were pointing toward a central lesion. Clinical and video test of skew offered little additional diagnostic value compared to other diagnostic tests such as the head impulse test and nystagmus test. Video test of skew could aid to quantify skew in the ED setting in which neurotological expertise is not always readily available.

**Supplementary Information:**

The online version contains supplementary material available at 10.1007/s00415-021-10692-6.

## Introduction

Skew deviation results from a dysfunction of the graviceptive pathways in acute dizziness leading to vertical diplopia due to vertical ocular misalignment [1]. It is considered as a central sign in patients with an acute vestibular syndrome (AVS) [2]; however, the prevalence of skew is not well known. Vertical eye deviation usually does not occur in isolation, but is accompanied by ocular counter-roll and associated head tilt in dizzy patients. This triad is known as ocular tilt reaction (OTR) [3]. Although skew deviation is predominately seen in patients with central lesions, peripheral causes of vertigo have also been reported in the literature [4]. However, skew deviation is reported to be smaller in peripheral lesions than in central causes [5].

Patients with AVS often do not mention vertical double vision because other symptoms, such as rotatory vertigo, nausea, and vomiting are more prominent in the acute state. It is therefore important that emergency physicians actively look for skew deviation, which is also integrated in the standardized three-step test ‘HINTS’ (Head-Impulse-Nystagmus-Test-of-Skew) [6]. Skew deviation can be clinically assessed at the bedside using the cross-cover or alternating-cover test (aka as test of skew). This test can be challenging in the presence of spontaneous nystagmus and needs expertise, which may often not be available in smaller community hospitals.

Nowadays, the use of video-oculography (VOG), assists physicians to quantify eye movements [7]. VOG is already used for the quantification of the head impulse test [8]; however, there are no reports about skew quantification by VOG in the emergency department (video Test of Skew, vTS).

In this prospective cross-sectional study, we sought to quantify eye misalignments in healthy subjects and to determine the prevalence of skew deviation in patients with AVS. We further assessed the diagnostic accuracy of clinical Test of Skew (cTS) versus video test of skew (vTS) for predicting stroke in the emergency department.

## Materials and methods

### Healthy subjects

We included 58 healthy subjects aged from 21 to 77 years without prior vestibular problems. A prerequisite for being considered healthy was a normal video head impulse test and a negative history of vertigo. We report normative data segregated by age decades including a minimal dataset of ten subjects per age group.

### Patients with acute vestibular syndrome

We enrolled AVS patients who met the inclusion criteria such as continuous dizziness, associated with nausea or vomiting, head-motion intolerance, new gait or balance disturbance, and nystagmus as part of a prospective cross-sectional study of patients seen in the emergency department (ED) (DETECT—Dizziness Evaluation Tool for Emergent Clinical Triage) between 07/2015 and 04/2020. We excluded patients younger than 18 years, if symptoms lasted < 24 h or if the index ED visit was > 72 h after symptom onset. We also excluded patients with previous eye movement or vestibular disorders. A neurootologist with an average of two years experience in the field performed a physical examination with clinical HINTS assessment, Caloric Testing, and video-TS testing in all enrolled patients. All patients received an acute MRI either within 48 h in the ED or a second, delayed MRI, if based on clinical grounds there was no acute MRI indicated or if the first acute MRI was non-diagnostic. The delayed MRI served as a gold standard for stroke detection. Patients with a negative MRI and either a pathological head impulse test or pathological caloric test were diagnosed as acute unilateral vestibulopathy (AUVP)/vestibular neuritis. Additionally, we collected information on age and gender.

We recorded the vertical ocular misalignment using a VOG device (EyeSeeCam, Munich) with an infrared video camera, and a frame rate of 220 Hz. The VOG device was calibrated by projecting dots on a TV screen or a tablet with a predefined distance (Tablet: Distance eyes to target: 260 mm, Target Size: 4 mm, Luminosity: 6.17 Lux, Angular size: 0.89 degrees. TV Screen: Distance eyes to target: 55 cm, Target Size: 5 mm, Luminosity: 11.8 Lux., Angular Size: 0.23 degrees). The vertical ocular misalignment was tested by fixating a dot displayed in the center of a TV screen or tablet. We used color-filtered glasses on both eyes (red filter for left eye and blue filter for right eye). The color filters allowed for a monochromatic view of the target dot, which changed periodically every 2 s from red to blue and vice versa. We maintained a standardized upright/ vertical head position using a chin rest avoiding any head tilt. Skew deviation was quantitatively reported in degrees (eye position) or converted into diopters. We report here the eye misalignment in degrees throughout the manuscript. Details of the applied method how to measure skew with VOG is reported elsewhere [9].

### Statistics

All statistics were reported using SPSS statistical software (IBM SPSS Statistics for Windows, Version 25.0. Armonk, NY: IBM Corp). We determined thresholds of physiological vertical ocular misalignments in healthy subjects using the 95th percentile. We used a non-parametric test (Kruskal–Wallis Test) to test for any effect of age and gender since the data were not normally distributed. Skew deviation exceeding the 95th percentile of the normatives was considered as a positive skew.

For the comparison of the vertical ocular misalignment between central and peripheral disorders, we only included patients with a confirmed stroke or AUVP and used a multivariate linear regression analysis taking into account the time interval between symptom onset and recording time.

We calculated the receiver characteristics curves (ROC) for VOG vertical misalignment and stroke prediction. The threshold for detecting clinically a positive skew was also determined by a ROC curve.

We used cross-tabulations to assess specificity (spec) and sensitivity (sens) for tests such as cTS and ‘HINTS’ with binary outcomes in predicting strokes. We evaluated stroke predictors using a binary logistic regression. The number needed to diagnose (NND) was calculated as follows: 1/(sens + spec-1). NND reflects the number of patients who need to be tested to correctly diagnose one person with stroke.

## Results

**Fig. 1:**
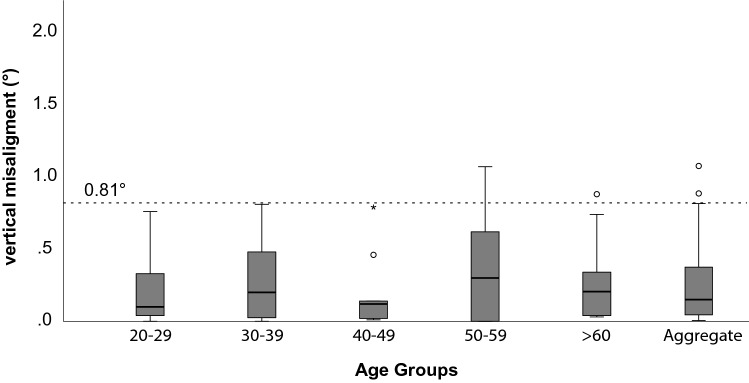
The box-plots whiskers and the outliers of vertical eye misalignment of normal subjects stratified by age groups. The circles represent the outliers (1.5 times of the interquartile range (IQR) above the upper quartile) and the asterisks the extreme outliers (3 times IQR above the upper quartile). The dotted line represents two standard deviations from the mean

Normative data from 58 healthy subjects are shown in table S1 and Fig. 1**,** stratified by age groups. Age (*p* = 0.887) and gender (*p* = 0.464) did not statistically affect test results. Based on our normative data, we considered a cut-off > 0.81 deg (95th percentile) as an abnormal vertical ocular misalignment.

We screened 1677 patients with acute dizziness of which 152 AVS patients aged between 20 and 91 (mean 55.67y) were enrolled. Out of 152 patients, 58 were diagnosed with AUVP (mean age 54y + / − 15.7y) and 27 patients with vestibular strokes (mean age 62.1y + / − 15.9y). Figure 1S (Appendix) shows a flow diagram with all screened patients, inclusions, and exclusions of dizzy patients. We analyzed data from 77 patients (aged between 20 and 88, mean 56.58y + / − 15.9y) with a diagnosis of stroke or AUVP and a valid vertical ocular misalignment measurement (53 with AUVP and 24 with stroke). Mean interval time from symptoms onset to VOG recordings was 29.7 h and, ranged from 1 to 74 h without statistically significant difference in both groups (*p* = 0.075). In addition, nystagmus intensity under fixation with gaze straight ahead was not correlated to the grade of skew deviation in light (Pearson Correlation, *p* = 0.489) and in darkness (*p* = 0.886).

The prevalence for skew deviation in AVS patients was 26% (20 out of 77). Skew prevalence in patients with AUVP was 24.5% (13/57) and 29.2% (7/24) in stroke patients.

**Fig. 2:**
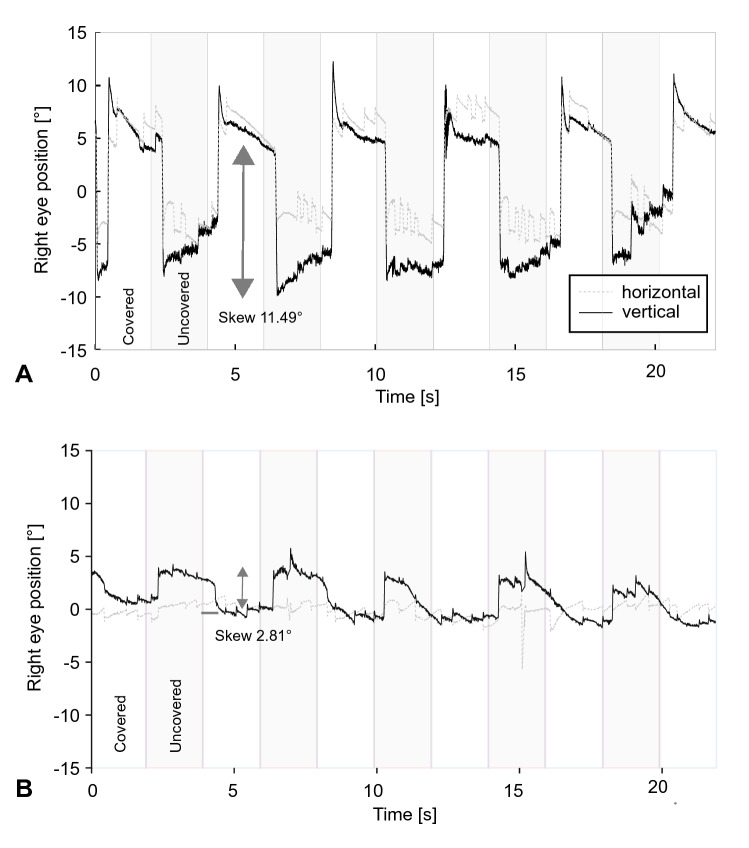
The eye recordings of VOG Test-of-Skew in a patient with acute stroke and an average of 11.49 deg skew (**A**) and in a patient with acute unilateral vestibulopathy and an average of 2.81 deg skew (**B**). The gray area represents the time that the eye is uncovered and fixing the target (y achse shows the eye position and x achse the time)

**Fig. 3:**
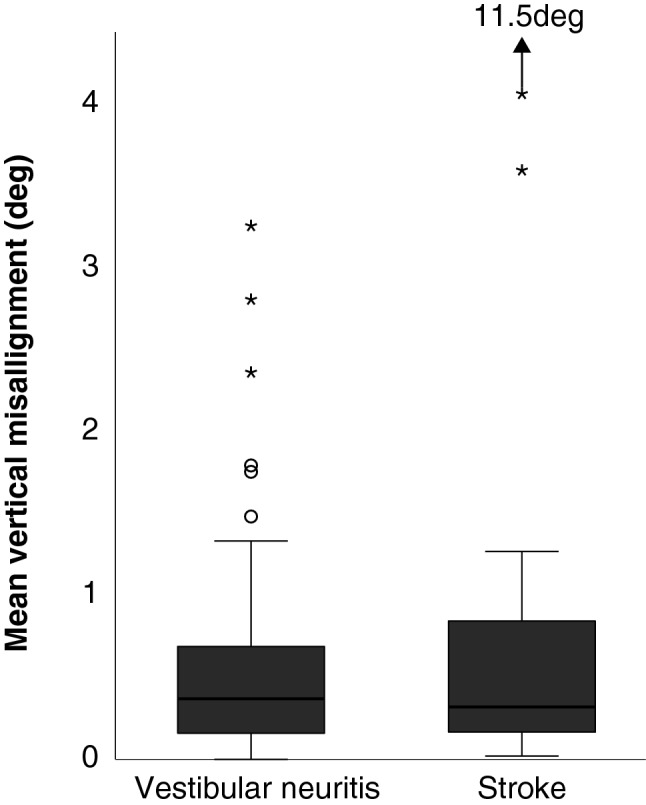
The box-plots whiskers and the outliers of vertical eye misalignment in patients with acute unilateral vestibulopathy and stroke. The circles represent the outliers and the asterisks the extreme outliers. Stroke group includes an outlier with 11.49° vertical misalignment, which is not represented graphically

Figure 2 shows the eye recordings of vTS in a patient with acute stroke (A) with 11.5 deg vertical ocular misalignment and AUVP (B) with 2.8 deg. Both patients reported vertical double vision. The stroke patient also presented with a laterodeviation, which is considered as a central sign [10]. Figure 3 shows the box-plots whiskers and the outliers of vertical ocular misalignment in patients with AUVP and stroke. There was statistically no difference in skew deviation between AUVP (median 0.37 deg + / − SE 0.10 deg, range from 0 deg to 3.26 deg) and stroke (median 0.32 deg + / − SE 0.48 deg, range from 0.02 deg to 11.49 deg) (*p* = 0.184), however, all patients (*n* = 2) with skew larger than 3.3 deg were found to be strokes (see extreme outliers, Fig. 3). The threshold for physicians to discern a vertical skew clinically was found to be at 3 deg deviation (5.25 prisms). Only six patients with skew deviation less than 3 deg reported double vision. One of them had a vestibular neuritis. Table S3 shows the characteristics of patients with a stroke. The clinicians performing the test of skew clinically had a very low sensitivity of 15% but a high specificity of 98.2% to detect skew greater than 0.81 deg.

**Fig. 4:**
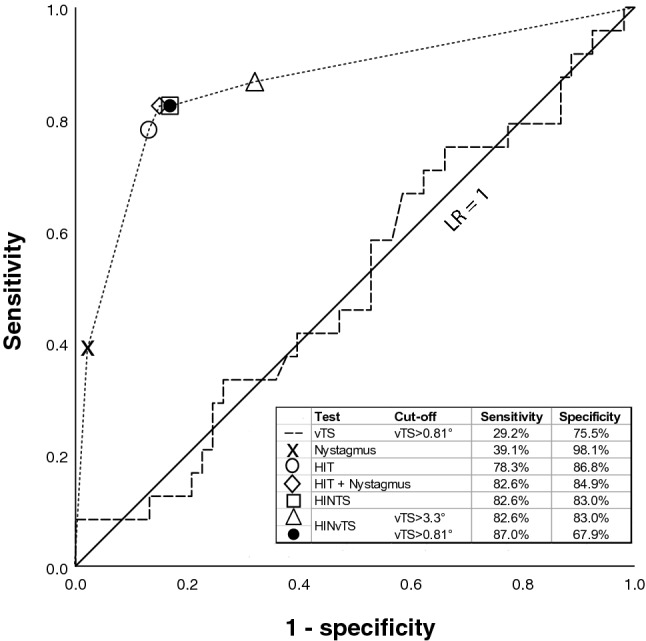
The sensitivity and specificity of ‘HINTS’ and its combinations: clinical head impulse alone, clinical head impulse and nystagmus combined, clinical ‘HINTS’ (three-step test) and clinical HINTS with integrated video test of skew

We did not find a significant discrimination cut-off of skew deviation between AUVP and strokes (AUC = 0.505, *p* = 0.943, CI 0.365–0.645, Fig. 4). The receiver operator characteristics curve (ROC) followed the depicted diagonal line representing a likelihood ratio of 1.

**Fig. 5:**
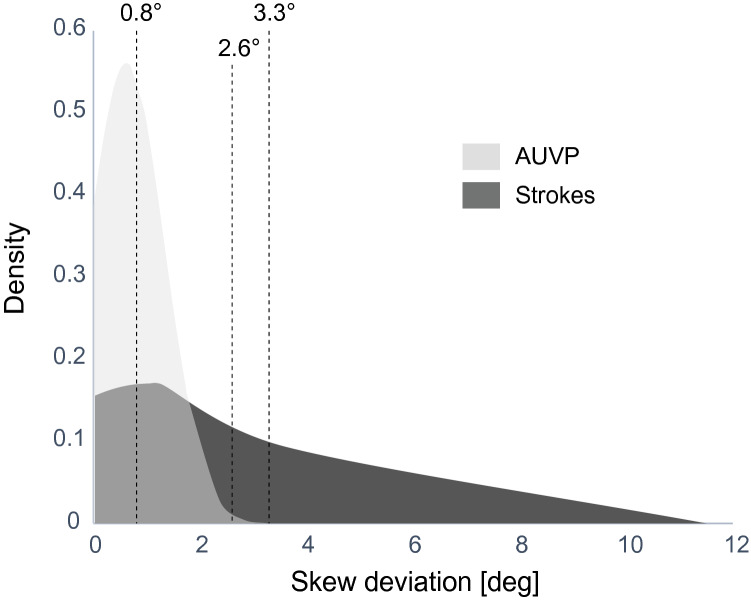
A density plot for skew deviations in patients with AUVP versus strokes. The vertical dotted lines illustrate the chosen optimal cut points to discriminate AUVP from stroke. A lower cut-off (0.81°) favored the sensitivity for stroke diagnosis while a higher cut-off (3.3°) favored the specificity for stroke detection

The sensitivity of a clinical test of skew to detect a stroke was 12.5%, the specificity 98.1% and its accuracy 71% (Cross table S2). The overall sensitivity in discriminating strokes with video test of skew (vTS), when vertical ocular misalignment was > 0.81 deg (arbitrary cut-off based on positive skew from normative data), was higher at 29.2% with a specificity of 75.5% (Fig. 4). The accuracy of vTS was 61%. Figure 5 shows a density plot for skew deviation in patients with AUVP and strokes. We chose additional skew deviation cut-offs (Fig. 5) for stroke detection either based on the AUVP population distribution (two standard deviations from the mean) or, being less conservative, the maximum skew value for AUVP (> 3.3°). These cut-offs resulted in a higher specificity of 96.2% for stroke detection (Cut-off > 2.6°) and 98.1% (cut-off 3.3°) for stroke detection. Sensitivity remained low at 8.3% for both limits.

Figure 4 shows the overall HINTS sensitivity and specificity in detecting strokes including cTS or vTS. It also shows the sensitivity of the head impulse alone or in conjunction with the assessment of nystagmus direction. Overall, clinical HINTS sensitivity was 82.6% and specificity 83%. The application of VOG for skew deviations with 0.81 deg as cut-off together with the other clinical steps of the HINTS protocol yielded the highest sensitivity of 87% while sacrificing specificity (67.9%, Fig. 5). The number needed to diagnose (NND) was 1.5 for clinical HINTS and 1.8 when we applied vTS. However, when we used 3.3 deg as a cut-off, the sensitivity, specificity and NND remained the same as for clinical HINTS. The application of VOG for skew detection with a cut-off > 2.61 deg resulted in a HINTS sensitivity of 79.2% and a specificity of 81.1%.

In the multivariate analysis, we found that the effect of vertical misalignment for a stroke was not statistically significant (*p* = 0.619), whereas the effect of age (*p* = 0.008) was statistically significant. Gender showed also no effect (*p* = 0.301).

## Discussion

We found a high prevalence of skew deviation in AVS patients with an estimate of one in four patients showing vertical eye misalignment. The prevalence was high in both, peripheral and central causes of dizziness. While there was a significant overlap between the two studied cohorts, we found large skew deviations (greater than 3.3 deg) as a stroke indicator. Skews greater than 3 deg could be discerned by clinicians.

VOG recordings of the vertical ocular misalignment is a convenient way to quantitatively estimate skew deviation in AVS patients. The test of skew alone, regardless of the measuring method (cTS vs. vTS), had an overall low sensitivity and a moderate accuracy to predict a stroke.

Although in the literature, there are many case reports [11–14] of skew deviation as a rare sign in peripheral deficits, to our knowledge, there is no study evaluating the prevalence of skew measured with VOG goggles in AUVP patients. In our study, the prevalence of a pathological vertical ocular misalignment was almost identical in both, AUVP and vestibular strokes. Kattah et al. [6] reported a lower prevalence of skew (tested with prism cover test) in peripheral AVS patients (4%), and an almost similar prevalence in central AVS patients (25%). One reason for these discrepancies could be a selection bias due to a relative low proportion of peripheral AVS in their study compared to ours. Carmona et al. in 2016 [15] also reported only four patients with skew (tested with the alternate cover test) out of 72 AUVP which is in line with our study when only our results of the clinical alternate cover test are considered. Since most of the AVS patients had a skew lower than 3 deg, which is not detectable by a human eye when nystagmus is present, the detection of skew in previous studies was probably underestimated. In addition, superior nerve division neuritis elicits a mixed horizontal/torsional nystagmus with a small vertical component, which can be falsely interpreted as skew. It is, however, questionable whether such small vertical fast phase movements would be discernable without VOG. We did not find skew differences between both cohorts (AUVP vs. stroke) with regard to gender. We found, however, differences in age between both groups, which is in agreement with other studies who report that patients with strokes are usually older [16].

Albeit we have shown that skew is more common than reported in the current literature on strokes and AUVP, we have not found a peripheral case with skew more than 3.3 deg (5.8 diopters). That means that a patient with a very large skew deviation (> 3.3 deg) is more likely to have a stroke. Such large skews are also clinically detectable and there is no absolute need for a VOG device; however, this holds only true for experts trained in eye movement assessment. This finding cannot be generalized to emergency physicians or non-experts. A VOG device, however, enables physicians to measure skew quantitatively and in a standardized way regardless of the skills of the examiner [17, 18].

Previous studies [19] used a semi-quantitative method for skew detection and assessed a high proportion of patients with stroke in their cohort. Thus, they reported a higher specificity.

The most accurate test for detecting a stroke in AVS patients was the head impulse test. This finding is consistent with previous studies [20]. Moreover, we found that the test of skew included in the ‘HINTS’ battery did not increase the diagnostic accuracy significantly but conversely increased the number of false positive results. This is due to the high prevalence of skew in patients with AUVP. The ‘HINTS’ protocol was reported to be better than an initial (acute) MRI with a sensitivity of 100% and specificity of 96% [6]. Our clinical diagnostic accuracy was lower possibly due to the 2 years of neurotological experience of the examiner compared to experts in the field.

There was no discrimination cut-off for diagnosing stroke based on vTS alone, however, taking an arbitrary cut-off of 0.81° (positive skew based on normative data), we demonstrated an increase in sensitivity (29.2%) compared to the low sensitivity of the clinical test (12.5%).

The application of HINTS with vTS increased the test sensitivity by 4%points, however, its specificity decreased by 17%points. The number needed to diagnose (NND) for clinical skew was 1.5 vs. 1.8 for vTS. This means that the number of patients needed to test for a correct diagnosis (true positive and true negative patients) was increased with vTS resulting in an increase in neuroimaging. As a consequence, we need to perform additional MRIs to detect a stroke patient which increases costs but this test potentially reduces the number of diagnostic errors [21]. vTS offered no additional diagnostic value but could still be helpful for non-experts, for the standardization and quantification of measurements.

### Limitations

This is the first large study in which both clinical and quantitative vertical ocular misalignment were assessed and compared. Future large-scaled studies with AVS patients and quantitative skew deviation measurements are needed to confirm that AUVP does not produce large skews greater than 3.3 deg.

Skew deviation could potentially also occur as a crosstalk between horizontal and vertical eye movements e.g., due to imprecise calibration. Other caveats include further potential test bias such as an incomplete binocular fusion (due to the dark environment and near target), the monocular recording and the decompensating phoria as a consequence of spontaneous nystagmus. Nevertheless, we showed that nystagmus intensity was not correlated to the grade of skew deviation. Equally, we also did not test visual acuity with both eyes which theoretically can lead to eye drifts and deviations on monocular fixation, however, we ensured that all patients were able to fixate the target point.

### Clinical implications

Clinical Test of Skew had a low sensitivity but high specificity to detect stroke provided that skew was larger than 3.3 deg. This test, however, cannot be used as a stand-alone test but is rather meant to be used as part of the HINTS protocol in patients with AVS. Overall, test of skew did not accurately discriminate AUVP from stroke regardless whether it was detected clinically or with VOG. Especially for clinicians lacking specialized training in oculomotor examination, vTS can help standardize an otherwise difficult exam and perhaps even establish an objective numerical cut-off in the future.

Furthermore, the vTS can serve as a diagnostic tool in an outpatient setting, when studying disease progression and compensation. Skew deviation usually resolves after days and might be used as a clinical sign of compensation of static vestibular function [22]. In addition, any information related to ocular alignment plays an important role to rehabilitation and large skew deviations can be a possible barrier [1].

## Conclusions

Contrary to prior knowledge, skew deviation proved to be more prevalent in patients with AVS and occurred in every forth patient with acute unilateral vestibulopathy. Thus, it was not necessarily a pathognomonic sign of central disease. Large skew deviations (> 3.3 deg), however, are a potential stroke indicator. Clinical and video test of skew offered little additional diagnostic value in detecting vestibular strokes compared to other superior diagnostic tests such as the head impulse test and nystagmus test. Video test of skew could aid to quantify skew in the ED setting in which neurotological expertise is not always readily available.

## Supplementary Information

Below is the link to the electronic supplementary material.

Supplementary file1 (PDF 359 KB)

## Abbreviations

AVS: Acute vestibular syndrome
AUVP: Acute unilateral vestibulopathy
HINTS: Head-Impulse-Nystagmus-Test-of-Skew
vTS: VOG Test of Skew
ED: Emergency department
