# Supplementary material for: Acute vestibular syndrome: is skew deviation a central sign?
Source: J Neurol. 2021 Jul 9;269(3):1396–403. doi: 10.1007/s00415-021-10692-6 (PMC8857098; doi:10.1007/s00415-021-10692-6)

Appendix

Table S1: Mean Skew Deviation relative to age.

Age Group	N	Minimum	Maximum	Mean	Standard Deviation (SD)	Upper Limit (Mean + 2 SD)
20-29	16	0.00	0.76	0.21	0.22	0.66
30-39	12	0.00	0.81	0.29	0.30	0.89
40-49	10	0.01	0.79	0.19	0.25	0.68
50-59	10	0.00	1.07	0.35	0.36	1.08
>60	10	0.03	0.88	0.29	0.30	0.89

Table S2: Frequency table for video and clinical test of skew.

			Stroke		
			No	Yes	Total
VOG TS*	No	N	40	17	57
		%	75.5%	70.8%	74.0%
	Yes	N	13	7	20
		%	24.5%	29.2%	26.0%
Clinical TS	No	N	52	21	73
		%	98.1%	87.5%	94.8%
	Yes	N	1	3	4
		%	1.9%	12.5%	5.2%
Total		N	53	24	77
		%	100.0%	100.0%	100.0%

*Cut-off for positive skew deviation: >0.81deg

Table S3: Characteristics of stroke patients

Gender	Age	Skew deviation (deg)	SPV of SN (deg/sec)*	Additional neurological signs	NIH Score	Lesion Site	Lesion Side	Hyper-tropic eye
m	87	0.41	0.77	No	0	Medulla	left	N/A
m	69	0.09	3.46	No	0	Cortical	left	N/A
f	57	0.27	0.36	Pathological left finger pointing test, GEN	2	Medulla, Pons, Cerebellum	bilateral	N/A
m	51	0.85	1.88	Quadrant anopsy, minor facial nerve palsy, GEN	4	Medulla, Pons, Midbrain, Cerebellum, Supratentorial WM	bilateral	left
m	75	0.14	0 **	GEN	0	Cerebellum	left	N/A
f	34	0.20	0.36	No	0	Cerebellum	bilateral	N/A
m	71	3.60	0.83	Light anisocoria, double vision, GEN	1	Cerebellum	right	right
f	65	0.84	5.29	Pathological left finger pointing test, tongue deviation to the left, GEN	3	Cerebellum	left	right
m	52	0.48	0.51	No	0	Supratentorial WM	right	N/A
m	61	1.04	1.38	Light anisocoria, GEN	0	Cerebellum	left	right
m	70	0.68	0.71	Pupillary reflex bilaterally, accomodation pathological right, pathological finger pointing test, double vision	3	Midbrain	right	N/A
m	71	0.21	0.78	Mild dysarthria, GEN	1	Pons	left	N/A
f	39	0.07	0.11	No	0	Supratentorial WM	right	N/A
m	73	0.32	2.11	Limb ataxia bds	2	Supratentorial WM, Cortical	right	N/A
m	74	0.52	1.49	Partial gaze palsy, limb ataxia, double vision	2	Medulla, Cerebellum	right	N/A
f	70	0.23	2.82	Mild dysarthria, limb ataxia, GEN	2	Cerebellum	right	N/A
f	74	0.11	2.23	No	0	Cerebellum	left	N/A
m	57	0.23	0.69	Partial gaze palsy, limb ataxia, mild dysarthria, double vision	3	Medulla, Cerebellum	bilateral	N/A
f	47	1.27	1.49	Severe to total sensory loss, GEN	2	Medulla	right	left
m	71	0.05	1.32	Double vision	0	Pons	right	N/A
f	71	0.02	0.04	Limb ataxia bds., double vision	2	Pons, Cerebellum	left	N/A
m	65	0.32	0.58	Pathological right finger pointing test	1	Medulla	right	N/A
m	62	0.97	9.17	No	0	Medulla, Cerebellum	left	right
m	75	11.49	0.31	Double vision , GEN	0	Cerebellum	right	right

*Gaze straight ahead without fixation

**0 °/s SPV because of gaze evoked nystagmus

Figure S1. Flow chart

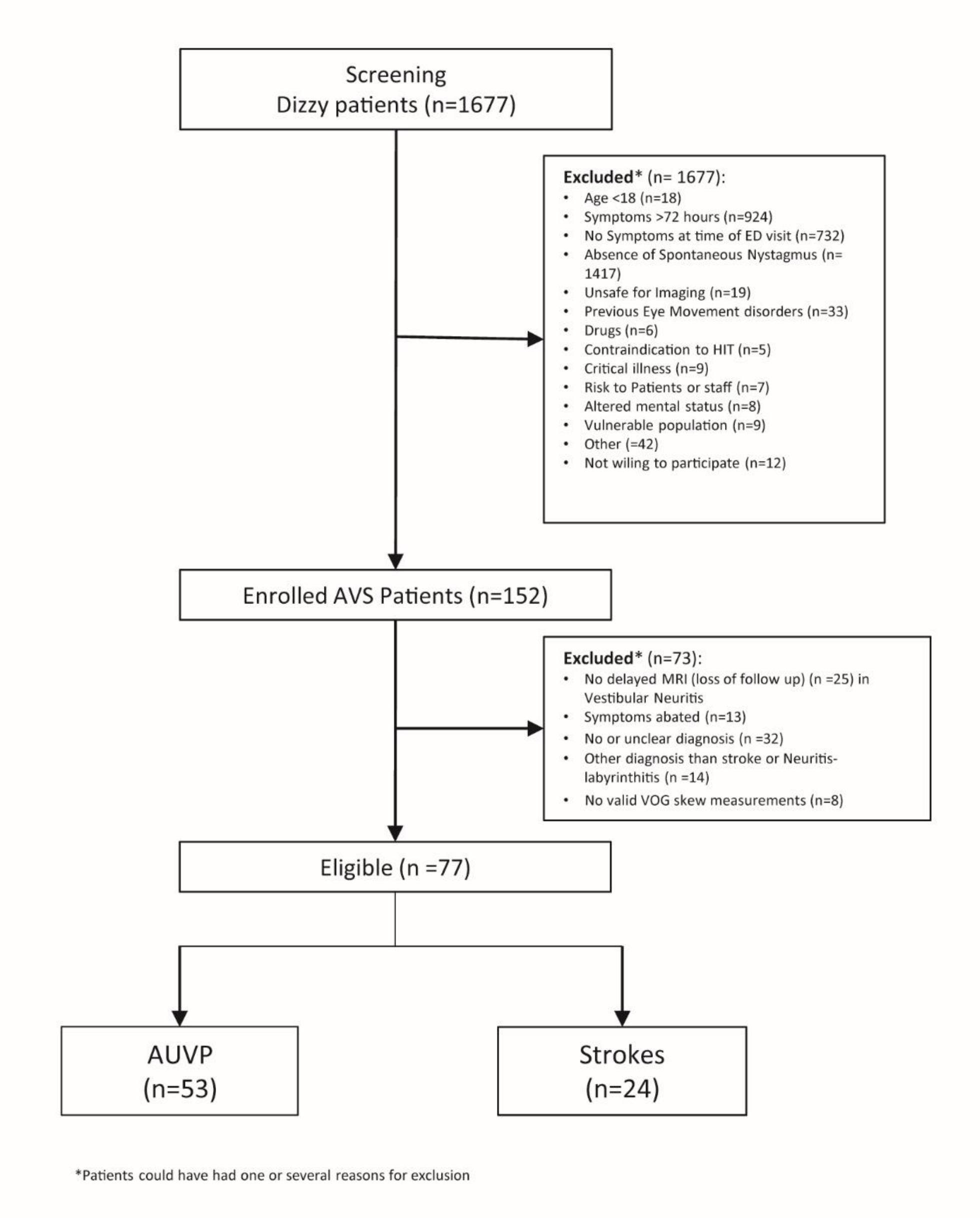

Supplement: Supplementary file 1 — Supplementary file1 (PDF 359 KB) [file 415_2021_10692_MOESM1_ESM.pdf]
